# Legislation

**Published:** 1897-11

**Authors:** 


### LEGISLATION.

*California.*—An act to regulate the practice of veterinary medicine in this State provides for the creation of an examining board, to consist of five duly qualified veterinarians, who are compensated out of the fees and forfeits which may accrue from the applicants for examination, but in no case is such remuneration to be taken from the State Treasury, and all surplus over and above these expenses is turned over to the State Treasurer. The fee for a graduate with a diploma from a recognized school is five dollars, and that of a non-graduate is ten dollars, which is forfeited should the examination not prove satisfactory to the board ; non-graduates, however, not to be examined after December, 1893. The meetings of the board are to take place every six months in San Francisco and Los Ángeles alternately. All licenses must be displayed prominently in the offices of practitioners, and a true copy filed in the County Court. Should this not be done within six months after the recepit of the license the offender is deprived of his license, unless he pays the sum of twenty-five dollars for the offense. Any infringement of this law is punishable by a heavy fine or imprisonment or both.

An ordinance has been issued by the County of Sacramento regulating the health of domestic animals and the sanitary condition of dairies, slaughter-houses, markets, and other places from which the food-supply is distributed. This ordinance provides that any animal having a contagious disease is to be reported to the veterinary inspector, and, after being notified by the inspector, the animal to be put to death by its owner. Any meat which has become putrid or unfit for use in any way shall not be sold or given away ; this also applies to the milk from diseased cattle. A thorough, sanitary cleanliness is to be maintained in the markets, slaughter-houses, or any place where milk and meat for consumption are kept. A violation of this ordinance is deemed a misdemeanor, and is punishable by fine or imprisonment or both.

An ordinance creating the office of veterinary inspector has also been adopted by the same county. He is empowered to investigate reports of contagious or infectious diseases in animals, and to order the animals destroyed if necessary ; also to enforce thorough sanitary conditions in such places as are used for the storage of meat- and milk-supplies. He must submit to the board of supervisors a detailed report of the examinations and tests made by him. He is to be supplied with an assistant or deputy, and such materials for carrying out the duties of his office as the exigencies of the situation demand.

*District of Columbia.*—The milk regulations for this district are very rigid, and were incorporated under an act to regulate the sale of milk in the District of Columbia, approved in 1895. This act provides that no dairy farms shall be maintained without a permit from the health officer of the district, who shall, after receiving application in due form, thoroughly inspect the premises before issuing the permit, which may be suspended at any time when danger to health is imminent from the exposure of the animals to any infectious or contagious disease. Any person desiring to send milk into the district for sale and consumption must obtain a permit for the same, and the application must be accompanied by a sworn statement of the physical condition of the cattle supplying the milk. All milk is to be tested by the Babcock method. Strict cleanliness, good drainage, ventilation, and water-supply are insisted on by the health officer, and enforced. Any milk from which any cream has been taken must be labelled "skim-milk," and sold as such. All milk-wagons must have on them the owner's name, the number of the permit, and the location of the dairy from which the milk is supplied. All analyses must be in the presence of two witnesses, one of whom may be the owner of the milk. Violations are punishable by fine or imprisonment, or both, and in case of a second offense the permit is withdrawn for six months.

